# A first in disease trial of the safety, tolerability, and anti‐seizure effects of ES‐481 in drug‐resistant epilepsy

**DOI:** 10.1002/epi4.70294

**Published:** 2026-06-18

**Authors:** Emma C. Foster, Lyn Millist, Jack Germaine, Stephanie Chamorro, Caitlin Roberts, John‐Paul Nicolo, Sean Hosking, Piero Perucca, Saul Mullen, Heather Robinson, Rebecca Wharrie, Brittany Walsh, David Reutens, Kimberley Irwin, Hao Zeng, Robert Niecestro, Terence J. O'Brien

**Affiliations:** ^1^ Department of Neuroscience, School of Translational Medicine Monash University Melbourne Victoria Australia; ^2^ Department of Neurology The Alfred Hospital Melbourne Victoria Australia; ^3^ Department of Neurology The Royal Melbourne Hospital Parkville Victoria Australia; ^4^ Bladin‐Berkovic Comprehensive Epilepsy Program, Department of Neurology Austin Health Heidelberg Victoria Australia; ^5^ Department of Medicine (Austin Health) The University of Melbourne Heidelberg Victoria Australia; ^6^ The Royal Brisbane and Women's Hospital Herston Queensland Australia; ^7^ ES‐Therapeutics New York New York USA

**Keywords:** anti‐seizure medication, clinical trial, epilepsy, Phase 2a, seizures

## Abstract

**Objectives:**

ES‐481 is a novel potent and selective antagonist of TARP‐y8‐dependent AMPA receptors. We aimed to assess the potential efficacy, safety and tolerability, and pharmacokinetics of different doses of ES‐481 as an add‐on anti‐seizure medication (ASM) in adults with drug‐resistant epilepsy (DRE).

**Methods:**

This was a Phase 2A double‐blind, randomized, dose‐titration, cross‐over, placebo‐controlled trial followed by an open‐label extension (OLE) conducted across four Australian Comprehensive Epilepsy Centers. The primary outcomes were: (i) efficacy, measured via change in seizure frequency for each treatment week, (ii) safety and tolerability, and (iii) pharmacokinetics. Secondary endpoints were change in anxiety and depressive symptom scores.

**Results:**

22 participants were randomized, 17 (77.3%) completed the double‐blind treatment phase, and 16 entered the OLE study. Overall, based on seizure diaries, participants had 68%–80% improvement in seizure frequency on ES‐481 treatment, compared with 38%–49% on placebo treatment (*p* = 0.097). For the highest dose of ES‐481 treatment (75 mg bid), subjects had 80% improvement (90% confidence interval [Cl] 43%–97%), while participants on placebo for the corresponding week had 49% improvement (90% Cl 1%–74%) (*p* = 0.047). There was no significant difference in the reduction of seizure frequency at the lower ES‐481 treatment doses. There were no significant differences in change in frequency of EEG epileptiform/seizure discharges >3 s in duration between the ES‐481 and placebo treatments. There was no significance difference in serious treatment‐emergent adverse events between the groups (placebo 14.3% vs. ES‐481 4.8%) or treatment‐emergent adverse events of special interest (e.g., dizziness, drowsiness; placebo 19.0% vs. ES‐481 52.4%, *p* = 0.052). Treatment was well tolerated in the OLE for up to 36 weeks.

**Significance:**

ES‐481 in doses up to 75 mg bid was safe and well tolerated for up to 36 weeks in participants with DRE and demonstrated potential anti‐seizure efficacy compared with placebo at this dose.

**Plain Language Summary:**

This was a first‐in‐disease, proof‐of‐concept early‐phase trial of a novel anti‐seizure medication, ES‐481, in adults with a broad group of patients drug‐resistant epilepsy, who continue to have seizures despite treatment with currently available medications. ES‐481 was generally safe and well tolerated, and participants receiving the highest dose showed fewer seizures compared with placebo. These findings suggest ES‐481 may offer a promising new treatment approach for people with difficult‐to‐control epilepsy, supporting the need for larger future studies.

**Trial Registration Information:**

Registered at ClinicalTrials.gov ID: NCT04714996 (first submitted and met QC criteria Jan 14, 2021, first posted Jan 20, 2021) and Australian and New Zealand Clinical Trials Registry ID: ACTRN12621000033842 (registered January 15, 2021). First participant/first visit (assessment) occurred on January 29, 2021.

**Key points**

- There is an ongoing need for the development of effective anti‐seizure medications for people with drug‐resistant epilepsy (DRE).
- ES‐481 is a potent and selective antagonist of the TARP‐y8‐dependent AMPA receptor, highly expressed in the hippocampus and limbic system.
- This Phase 2a study found ES‐481 to be safe, well tolerated, and had potential anti‐seizure efficacy compared with placebo at 75 mg bid.

## 1 BACKGROUND

Epilepsy is a serious chronic neurological disorder characterized by recurrent, unprovoked seizures.^1^ One in three people living with epilepsy have drug‐resistant epilepsy (DRE).^2^ DRE is defined by the International League Against Epilepsy (ILAE) as ongoing seizures despite adequate trials of two or more appropriate anti‐seizure medications (ASMs).^3^, ^4^ DRE drives much of the global burden of epilepsy.^5^, ^6^ Although therapeutic interventions such as epilepsy surgery, neurostimulation, and dietary therapies may be appropriate in specific cases of DRE, there remains an ongoing need for the development of more effective ASMs for people with DRE.^7^

ES‐481 is a novel potent and selective antagonist of the transmembrane alpha‐amino‐3‐hydroxy‐5‐methyl‐4 isoxazolepropionic acid (AMPA) receptor regulatory protein (“TARP”)‐γ8‐dependent AMPA receptor, which is highly expressed in the hippocampus and limbic system. This targeted approach potentially reduces the likelihood of treatment‐emergent adverse events by minimizing more widespread effects on AMPA receptors in the CNS, including areas such as the cerebellum.^5^, ^8^ Preclinical toxicity studies identified the main dose‐related toxicology effects of ES‐481 are on the CNS and cardiovascular functions. In a Phase 1 single ascending dose clinical trial in healthy human volunteers, ES‐481 was well tolerated in doses up to 100 mg bid, with no participants having a serious adverse reaction or discontinuing the trial.^6^ Perampanel, which is a non‐selective AMPA receptor antagonist, is clinically approved as an effective ASM, but adverse effects are seen in a significant proportion of patients which can limit the effectiveness of the treatment.^9^, ^10^, ^11^ Here, we report the results of a Phase 2A trial that aimed to evaluate the potential efficacy, safety, and tolerability of different doses of ES‐481 as an add‐on ASM in adults with DRE.

## 2 MATERIALS AND METHODS

### 2.1 Trial design

This was a Phase 2A, double‐blind, randomized, placebo‐controlled study with cross‐over and an open‐label extension.

Eligible participants were randomized in a 1:1 ratio to either sequence 1 (ES‐481/Placebo) or sequence 2 (Placebo/ES‐481). Participants who completed the double‐blind treatment phase had the opportunity to participate in the open‐label extension phase for up to 36 weeks treatment, titrated to a dose that was deemed most suitable by the treating neurologist.

### 2.2 Eligibility criteria

The study was open to adults aged 18–70 years with DRE,^3^ who provided written informed consent to participate. Although ES‐481 targets γ8‐dependent AMPA receptors which are differentially expressed in the hippocampus and limbic structures, participants with a wide spectrum of epilepsy syndromes, including both focal and generalized DRE, were eligible for inclusion in the study to assess ES‐481's efficacy, safety, and tolerability across a broad range of patients in this early phase trial. Participants were taking a stable dose of ASMs for at least 4 weeks prior to entering the 28 days screening period. If they had a vagus nerve stimulator implanted, the stimulation settings had to be stable for at least 4 weeks prior to entering the screening period. During the screening period, participants had to experience at least four countable seizures, and there needed to be on average at least one interictal epileptiform discharge per hour on a 24‐h EEG recoding to ensure participants had a sufficiently “active” EEG for an effect of the treatments to be seen.

People who did not meet all the above criteria were excluded from the study. Additional exclusion criteria were pregnancy or breastfeeding, unwilling or unable to take adequate contraceptive precautions, significant comorbidities (e.g., diabetes, heart disease) or biochemical derangement, use of recreational drugs within the prior month and no commitment to abstain from these for the duration of the study, alcohol dependence, progressive neurologic disorder (e.g., brain tumor, multiple sclerosis, dementia), and planned epilepsy surgery within 6 months before or after enrolment in the study.

### 2.3 Settings and locations where the data were collected

Participants were enrolled and studied at four Australian Comprehensive Epilepsy Centers that were part of the Australian Epilepsy Clinical Trials Network (AECTN): Alfred Hospital, Austin Hospital, The Royal Melbourne Hospital, and The Royal Brisbane and Women's Hospital.

### 2.4 Interventions

Following the baseline 28‐day observation period, participants were randomized 1:1 to study drug‐ or placebo‐first groups. The first treatment period ran 28 days, with participants in the treatment group receiving four escalating doses of ES‐481 over a 4‐week period (week 1: 25 mg daily; week 2: 25 mg bid; week 3: 50 mg bid; week 4: 75 mg bid) and participants in the placebo group receiving matching placebo doses. Participants in all groups had a study visit, 24‐h continuous EEG, and pharmacokinetic testing prior to the start of each dose increment. This treatment period was followed by a 7‐day step down period and a further 7‐day wash out period, and the groups then crossed‐over. The second treatment period ran for a further 28 days, following the same pattern as for the first treatment period, followed again by a 14‐day step down and wash out period. This was then followed by an open‐label extension of 36 weeks, with dosing of ES‐481 during this period determined by the treating neurologist, up to a maximum dose of 75 mg bid.

#### 2.4.1 Primary outcomes

As a Phase 2A study, the three primary objectives were to: (i) evaluate safety and tolerability, (ii) estimate pharmacokinetics, and (iii) generate indicative efficacy data to inform subsequent larger scale randomized control trials.

##### Efficacy

The primary efficacy outcome of change in seizure frequency during ES‐481 treatment during the double‐blind phase compared with baseline was assessed via log‐transformed change in seizure frequency, as measured via patient or carer self‐report (seizure diaries). Only seizures with an observable motor component or with impaired consciousness were counted, which included focal impaired consciousness, focal to bilateral tonic–clonic, generalized‐onset tonic–clonic, tonic, atonic, and myoclonic seizures. Baseline seizure frequency was established via a 28‐day seizure diary. Seizure frequency during the double‐blind phase was assessed through seizure diaries at Days 1, 8, 15, 22, 28, 43, 50, 57, 64, and 70 timepoints. At these same timepoints, the change in the frequency of epileptiform/seizure discharges on a normalized 4–24 h EEG was assessed as an additional primary efficacy outcome, as well as representing part of the safety assessment (as described below).

50% reduction in seizure frequency from baseline was also calculated from the seizure diary data; this was a non‐primary efficacy outcome.

### 2.5 Epileptiform activity on the EEG

Participants underwent 11 ambulatory 24‐h EEG recordings throughout the study, including one during their 28‐day screening period. They attended their study site, were connected to EEG, were discharge home, and then returned the following day for disconnection. The recordings were sent to a central area for processing; identifiers including names, dates, and times of recordings were stripped from the raw recordings, and the recordings were randomly assigned to “boxes” of 10 for reading by a senior neuroscientist and board‐certified epileptologist. Recordings in which there were at least 4 h of technically satisfactory EEG recordings were included in the analysis. Events meeting criteria for International Federation of Clinical Neurophysiology epileptiform discharges were marked at point of onset and offset, by consensus opinion.^12^, ^13^ Epileptiform/seizure discharges of >3 s duration were utilized in the primary efficacy analysis. Separately, as part of an exploratory analysis, we compared self‐reported seizure counts (seizure diary) with frequency of EEG epileptiform/seizure events lasting >3 s.

### 2.6 Safety assessments

Safety assessments were performed at Days 1, 8, 15, 22, 28, 43, 50, 57, 64, and 70, and included screening for CNS and cardiovascular treatment‐emergent adverse events, vital signs, hematology and biochemistry laboratory tests, neurological and physical examinations, ECG, and EEG. Treatment‐emergent adverse events of special interest were recorded, including anxiety, ataxia, agitation, behavioral change, dizziness, drowsiness, mood swings, insomnia, somnolence, paresthesia, feeling abnormal, disturbance in attention, gait disturbance, and dysarthria. These were based on findings from preclinical (animal) and Phase 1 human studies.^6^ Serious treatment‐emergent adverse events were defined as life threatening events, persistent or significant disability/incapacity, resultant congenital anomaly or birth defect, requiring inpatient hospitalization or prolonged hospitalization, other medically important events, and death.

#### 2.6.1 Pharmacokinetics

Pharmacokinetics were assessed using compartmental methods to determine Tmax, AUCo‐t, AUCo‐inf T1/2, CL/F, and Vz/F at Days 1, 8, 15, 22, 28, 43, 50, 57, 64, and 70.

### 2.7 Secondary outcomes

Changes in anxiety and depression scores, measured via Hamilton Anxiety Rating Scale (HAM‐A) and Hamilton Depression Rating Scale (HDRS), respectively, were also assessed at Days 1, 8, 15, 22, 28, 43, 50, 57, 64, and 70.

The following changes were made to the protocol after the trial commenced:

- Expansion of inclusion criteria to allow for more flexible enrolment: increase cut‐off age from 65 to 70 years; interictal epileptiform discharges had to occur with an average frequency of approximately one (rather than at least one) per hour on EEG recording; and if there was a history of illicit substance use in the month prior to study enrolment, participants would still be eligible for enrolment if they committed to not take substances during the study.
- Removal of certain investigations that are not done routinely in Australia, and were not considered critical to assessing participant safety (blood urea nitrogen, gamma‐glutamyl transpeptidase, lactate dehydrogenase, and carbon dioxide);
- Removal of the Columbia Suicide Severity Rating Scale, as the Hamilton Anxiety and Depression Rating Scales were considered sufficient;
- Shortening the minimum acceptable duration of continuous EEG recording for inclusion in the analysis from 24 to 4 h, recognizing that EEG electrodes become dislodged during the 24‐h recording window in some patients;
- Inclusion of an open‐label extension phase to the study.

### 2.8 Sample size

As this was a first‐in‐disease study for ES‐481, no clinical efficacy data are available, and thus, no formal sample size calculation was performed. Phase 2A studies, such as this one, focus on generating efficacy and safety data to inform subsequent larger, later phase trials, and are not adequately powered to make conclusive statements regarding drug efficacy or secondary outcomes such as mood.

### 2.9 Interim analyses

No interim analyses were planned or performed.

### 2.10 Randomization

The randomization list was created independently by the Clinical Research Organization, Neurotrials Australia, using Proc Plan (a function in SAS) with default SEED. Since the number of blocks and block size were masked, no potential unblinding risks were identified. A four‐digit randomization number (for ES‐481/placebo dispensation) was initiated by the site staff and assigned to the participant. ZIFO RnD Solutions (PureCDM's statistical services provider) sent the live randomization list to the unblinded pharmacist at each site via email, who stored and dispensed the drug according to the protocol and randomization code.

### 2.11 Blinding

Blinding (masking used) was applied to people receiving the treatments, people administering the treatments, people assessing the outcomes, and people analyzing the data. The unblinded pharmacist dispensed the study medication for an individual participant dosing according to the protocol and randomization code. Study medications (ES‐481 or matching placebo) were provided as 25 mg capsules. Matching placebo capsules were also supplied by ES Therapeutics.

### 2.12 Statistical methods

Unless otherwise noted, continuous variables were summarized using number (*n*) of non‐missing observations, mean, standard deviation (SD), median, minimum, and maximum; categorical variables were summarized using the frequency count and the percentage of participants in each category.

In general, summary statistics (*n*, mean, SD, median, minimum, maximum, and, for all continuous parameters, the coefficient of variation) were presented by scheduled sample time for PK concentrations and PK parameters.

The primary endpoint of patient‐reported seizure frequency was not normally distributed and so underwent log‐transformation prior to being analyzed using a mixed model with repeated measures. The model included sequence, treatment, period, week, and treatment‐by‐week interaction as fixed effects, and log‐transformed baseline value as a covariate. Robust sandwich method was used to estimate the covariance matrix. The additional primary efficacy endpoint of normalized 4‐h EEG epileptiform activity was analyzed using mixed‐model repeated measures without log transformation. The normalized 4‐h EEG epileptiform activity was derived as 4 multiplied by the total number of epileptiform discharges >3 s duration reported by the independent blinded epileptologist, divided by the total hours of monitoring time that could last up to 24 h. The frequency of interictal EEG discharges (<3 s duration) was also compared as an exploratory endpoint. All participants had to have at least 4 h of analyzable EEG recordings for inclusion in the analysis.

The secondary endpoints of HAM‐A and HDRS were analyzed using the same mixed‐model with repeated measures without log‐transformation.

No imputation method was applied to the missing data in the efficacy analysis.

### 2.13 Standard Protocol Approvals, Registrations, and Participant Consents

This study was approved by Alfred Health's Human Ethics Research Committee (HREC number 688.20). Prospective written informed consent was obtained from all participants (or guardians of participants) in the study (consent for research).

Trial registered at ClinicalTrials.gov ID: NCT04714996 (first submitted and met QC criteria Jan 14, 2021, first posted Jan 20, 2021) and Australian and New Zealand Clinical Trials Registry ID: ACTRN12621000033842 (registered January 15, 2021). First participant / first visit (assessment) occurred on January 29, 2021.

### 2.14 Data Availability

Data not provided in the article because of space limitations may be shared (anonymized) at the request of any qualified investigator for purposes of replicating procedures and results.

The full trial protocol will be made publicly available at the conclusion of current commercial negotiations regarding funding for the next stage of clinical development of ES‐481, anticipated by 2026. In the meantime, a summary of the protocol may be accessed via the ANZCTR. Qualified investigators may contact the study team if there are specific protocol queries in the meantime.

This study is reported using the CONSORT reporting guidelines.^14^

## 3 RESULTS

### 3.1 Study participation

The first participant's first visit occurred on January 29, 2021 and the last participant's last visit occurred on June 29, 2023. A total of 30 participants were screened, of which 22 were randomized in a 1:1 ratio to either Sequence 1 (ES‐481/Placebo, with ES‐481 in Treatment Period 1, and Placebo in Treatment Period 2) or Sequence 2 (Placebo/ES‐481). All randomized participants received at least one dose of the study drug and were included in the safety analyses. Of the 17 participants that completed the study, 16 entered the open‐label extension phase. Overall, 5 participants withdrew from the study, 3 (13.6%) during placebo treatment due to treatment‐emergent adverse events, and 2 (9.1%) during ES‐481 treatment due to treatment‐emergent adverse events (*n* = 1) and participant request (*n* = 1). The primary analysis was based on the modified intention‐to‐treat (mITT) population and involved all participants who were randomized, took at least 1 dose of study drug, and had at least 1 post‐baseline assessment. The trial ended once all study visits had been completed.

**FIGURE 1:**
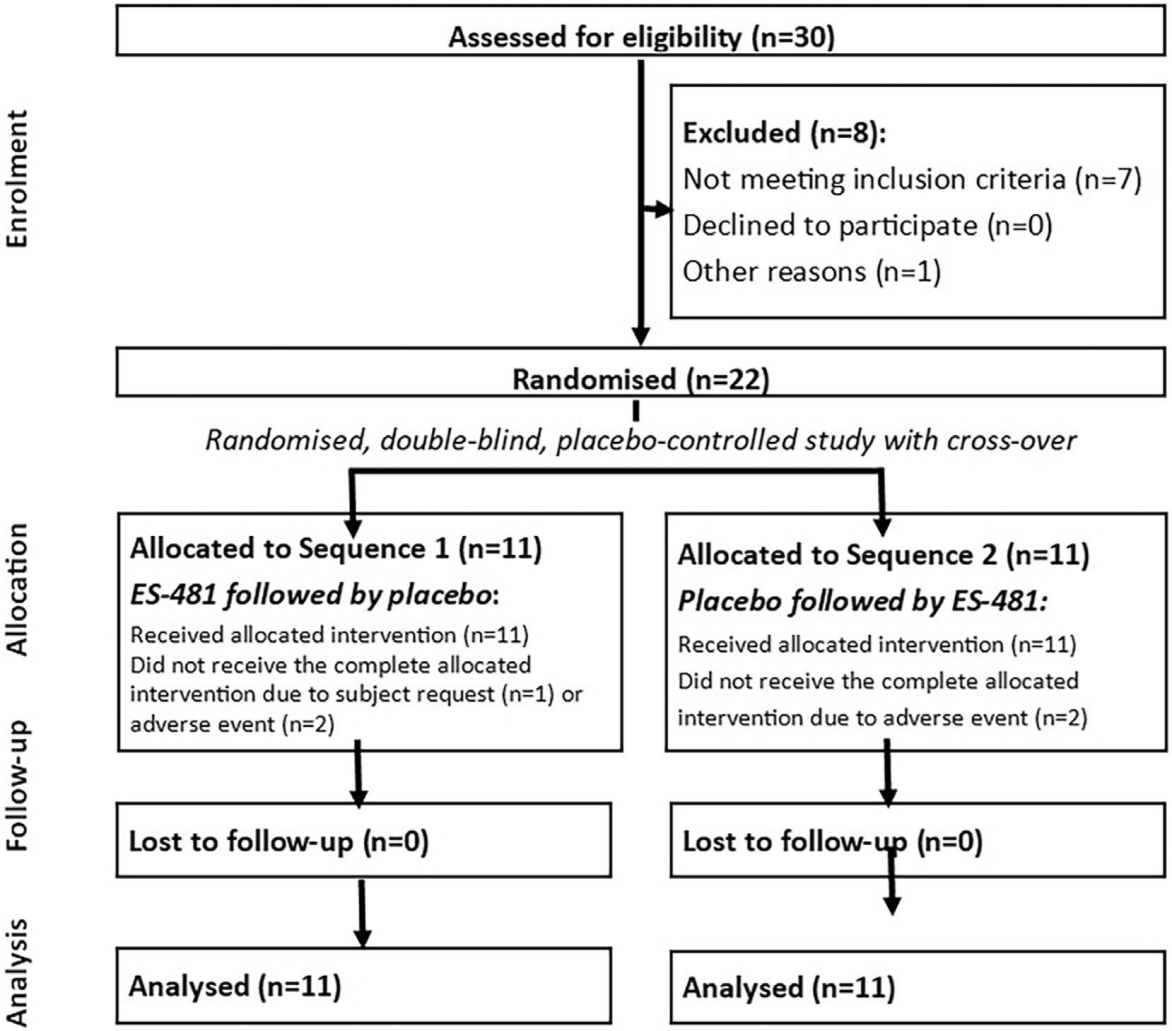
Participant flow diagram. All randomized participants received at least 1 dose of the study drug and were included in the safety analyses. Of the 17 participants that completed the study, 16 chose to continue in the open‐label extension.

Figure 1 displays the CONSORT flow diagram.

### 3.2 Baseline data

In the modified intention‐to‐treat (mITT) population, the median (SD) age was 37.5 years (15.15). Thirteen (59.1%) participants were male and 77.3% were white. Fifteen (68.2%) had focal epilepsy, 7 (31.8%) had generalized epilepsy, and they had a mean average of 27.4 patient‐reported seizures each week. All participants had at least one medical comorbidity. The baseline clinicodemographic characteristics were balanced between the two sequences, with no statistically significant differences between them.

Baseline characteristics of the mITT group are displayed in Table 1.

**TABLE 1:** Baseline demographic and clinical characteristics for each group.

Characteristic	ES‐481/placebo *N* = 11	Placebo/ES‐481 *N* = 11	Overall *N* = 22
Age (years)			
*n*	11	11	22
Mean (SD)	34.4 (18.30)	40.5 (11.21)	37.5 (15.15)
Median	26.0	37.0	34.5
Min, Max	18, 70	28, 57	18, 70
Sex, *n* (%)			
Male	5 (45.5)	8 (72.7)	13 (59.1)
Female	6 (54.5)	3 (27.3)	9 (40.9)
Ethnicity, *n* (%)			
Hispanic or Latino	1 (9.1)	0	1 (4.5)
Not Hispanic or Latino	8 (72.7)	10 (90.9)	18 (81.8)
Not reported	0	0	0
Unknown	2 (18.2)	1 (9.1)	3 (13.6)
Race, *n* (%)			
Black or African American	0	0	0
American Indian or Alaska Native	0	0	0
Asian	2 (18.2)	1 (9.1)	3 (13.6)
Native Hawaiian or Other Pacific Islander	0	0	0
White	8 (72.7)	9 (81.8)	17 (77.3)
Multiple	1 (9.1)	1 (9.1)	2 (9.1)
Other	0	0	0
Not reported	0	0	0
Epilepsy type, *n* (%)			
Focal	5 (45.5)	10 (90.9)	15 (68.2)
Generalized	6 (54.5)	1 (9.1)	7 (31.8)
Weekly seizure frequency at Baseline			
*n*	11	11	22
Mean (SD)	41.24 (69.241)	13.57 (7.327)	27.41 (50.091)
Median	11.08	11.03	11.05
Min, Max	3.5, 242.0	7.7, 32.7	3.5, 242.0
Concomitant anti‐seizure Medications, *n* (%)			
1 medication	1 (9.1)	0	1 (4.5)
2 medications	5 (45.5)	1 (9.1)	6 (27.3)
3 medications	2 (18.2)	7 (63.6)	9 (40.9)
4 medications	3 (27.3)	3 (27.3)	6 (27.3)

### 3.3 Outcomes

#### 3.3.1 Efficacy

##### Primary efficacy outcome—seizure diaries

**FIGURE 2:**
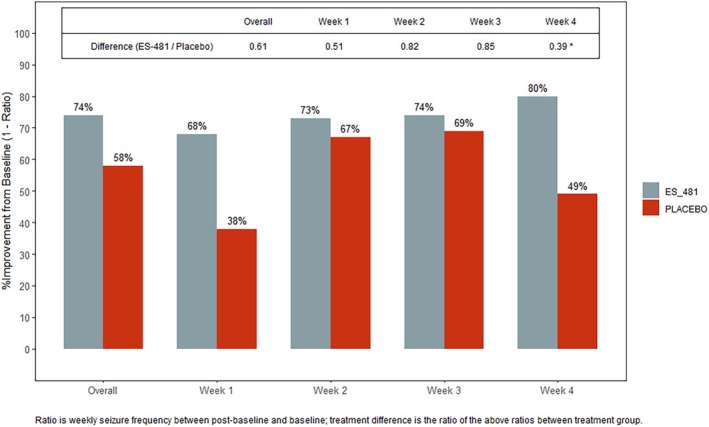
Primary efficacy outcome—change from baseline in weekly seizure frequency for the double‐blind treatment phase (modified intention‐to‐treat population). The ratio is the average weekly seizure frequency between post‐baseline and baseline; treatment difference is the ratio of the above ratios between treatment groups.

Based on self‐reported seizure frequencies recorded in seizure diaries, participants had an overall 68–80% improvement relative to baseline on ES‐481 treatment compared with 38–69% on placebo (*p* = 0.097). For the highest ES‐481 treatment dose (75 mg bid), participants had 80% improvement (90% confidence interval [CI] 43%–93%), while the corresponding placebo participants had 49% improvement (90% CI 1%–74%) (*p* = 0.047). Lower ES‐481 doses did not significantly differ from placebo in terms of change in seizure frequency. Table 2 displays the change from baseline in weekly seizure frequency for the double‐blind treatment phase, and Figure 2 displays the ratio between ES‐481 and placebo for weekly seizure frequency compared with baseline. Table S1 reports a summary of change from baseline in weekly seizure frequency in the double‐blind treatment phase. Figure S1 compares the ratio of improvement in seizure frequency from baseline in the placebo arms for Treatment Period 1 versus 2.

**TABLE 2:** Primary outcome—change from baseline in weekly seizure frequency for the double‐blind treatment phase (modified intention‐to‐treat population).

Visit	ES‐481 *N* = 22	Placebo *N* = 22	Difference (ES‐481—placebo)	*p*‐value
Overall				
LSMean of Log‐transformed Score (SE)	−1.35 (0.454)	−0.86 (0.405)	−0.49 (0.366)	0.097
Ratio^a^	0.26	0.42	0.61	
90% CI	(0.12, 0.57)	(0.21, 0.85)	(0.32, 1.15)	
Week 1				
LSMean of Log‐transformed Score (SE)	−1.15 (0.576)	−0.48 (0.367)	−0.68 (0.529)	0.104
Ratio^a^	0.32	0.62	0.51	
90% CI	(0.12, 0.83)	(0.34, 1.15)	(0.21, 1.24)	
Week 2				
LSMean of Log‐transformed Score (SE)	−1.29 (0.569)	−1.10 (0.665)	−0.19 (0.771)	0.402
Ratio^a^	0.27	0.33	0.82	
90% CI	(0.11, 0.71)	(0.11, 1.01)	(0.23, 3.00)	
Week 3				
LSMean of Log‐transformed Score (SE)	−1.34 (0.566)	−1.18 (0.633)	−0.16 (0.513)	0.376
Ratio^a^	0.26	0.31	0.85	
90% CI	(0.10, 0.68)	(0.11, 0.89)	(0.36, 2.01)	
Week 4				
LSMean of Log‐transformed Score (SE)	−1.62 (0.629)	−0.68 (0.399)	−0.94 (0.550)	0.047
Ratio^a^	0.20	0.51	0.39	
90% CI	(0.07, 0.57)	(0.26, 0.99)	(0.16, 0.98)	

*Note*: Participant‐reported seizure frequency is the weekly seizure frequency derived by the average of the non‐missing scores captured during the week multiplied by 7. The weekly frequencies are analyzed after log‐transformation, a value of 0.01 is imputed before taking the log when the frequency of seizures is 0 at a given week. The Mixed Model with Repeated Measures (MMRM) includes Sequence, Treatment, Period, Week, and Treatment‐by‐week interaction as fixed effects, and log‐transformed baseline as the covariate. Robust sandwich method is used to model the covariates. Geometric mean is calculated after anti‐log‐transformation that converts the results to the original scale. a: Ratio is calculated after anti‐log‐transformation of the LS Mean of log‐transformed score. For each treatment group, this is the ratio of weekly seizure frequency between post‐baseline and baseline; for the treatment difference, this is the ratio of the above ratios between treatment group (ES‐481 vs. placebo). One‐sided *p*‐value was calculated.

##### Primary efficacy outcome and safety analysis—EEG electrographic seizure activity

There were no significant differences in the change from baseline frequency of electrographic epileptiform/seizure events >3 s between ES‐481 and placebo treatments for the normalized 4‐h EEG, with both groups demonstrating reduced EEG discharges (ES‐481 vs. placebo: −2.13 vs. −2.19, *p* = 0.438). These data are summarized in Table S2. There were also no significant differences observed between the treatment groups for the frequency of interictal EEG discharges (i.e., <3 s duration).

##### Exploratory efficacy outcome—Seizure frequency responder rate

**FIGURE 3:**
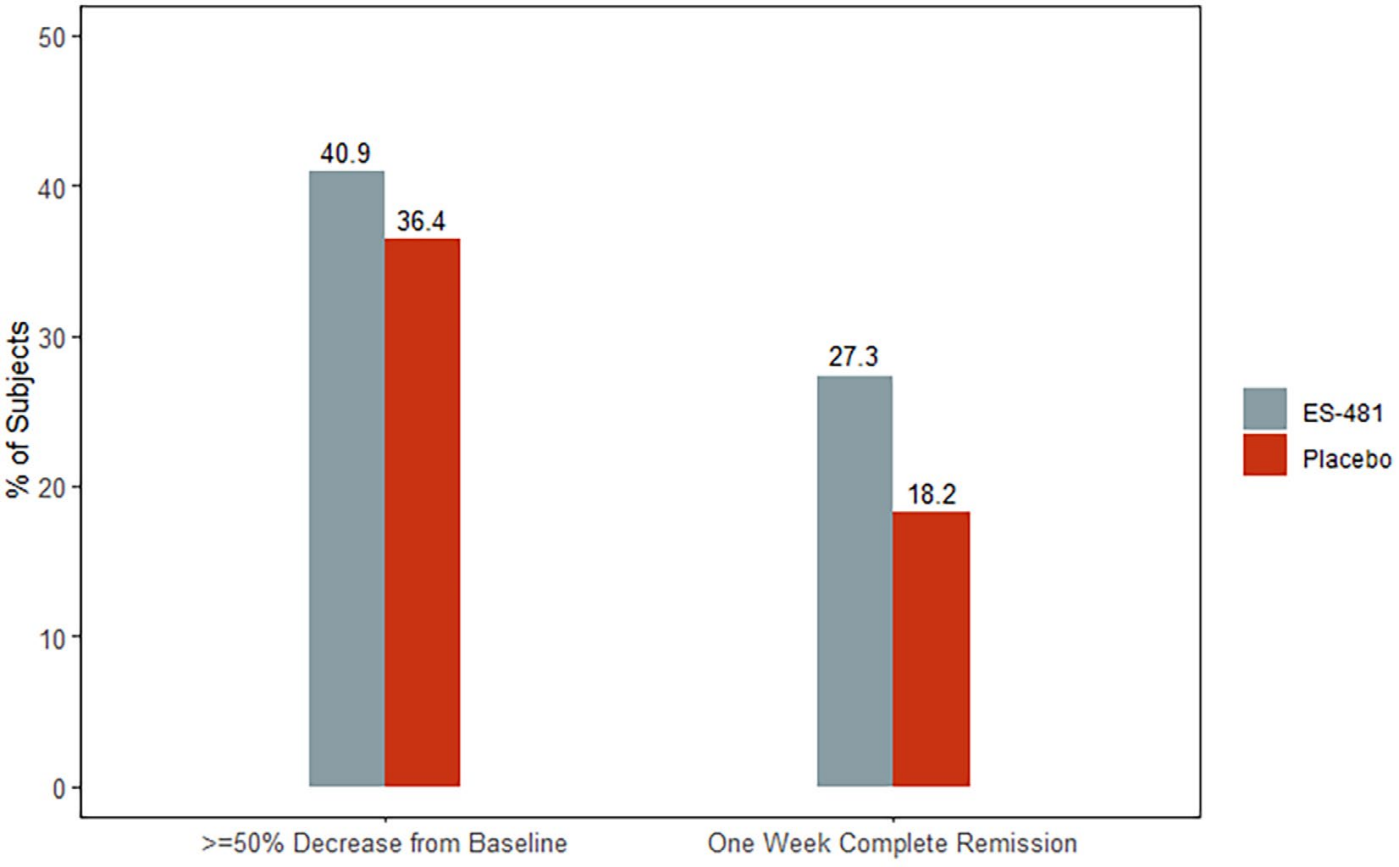
Non‐primary efficacy outcome—seizure frequency responder rates for the double‐blind treatment phase (modified intention‐to‐treat population). These findings were not statistically significant.

Seizure frequency responder rate (50% reduction in seizure frequency from baseline) was also higher, but not statistically significant, with ES‐481 treatment than with placebo. These efficacy outcomes are displayed in Figure 3.

##### Open‐label extension phase

During the open‐label extension phase, ES‐481 reduced the weekly seizure frequency relative to baseline by at least 70%, apart from Week 2 (60% reduction) and Week 5 (50% reduction). These data are reported in Figure S2.

#### 3.3.2 Safety assessments

The mean (SD) duration of exposure to ES‐481(3.9 [0.7] weeks) and placebo (3.7 [1.0] weeks) was comparable in the double‐blind treatment phase. The median duration of exposure to ES‐481 was 26.9 weeks in the open‐label extension phase.

In the double‐blind treatment phase, the incidence of treatment‐emergent adverse events was, in general, comparable between the two treatments (ES‐481 vs. placebo: 85.7% vs. 76.2%, *p* = 0.70). Four participants (19%) experienced treatment‐emergent adverse events leading to drug discontinuation, three while receiving placebo and one while receiving ES‐481. One participant experienced a serious adverse event on the ES‐481 treatment arm (4.8%) and three on the placebo arm (14.3%). No participants died during the study. Treatment‐emergent adverse events of special interest were more common while taking ES‐481 than placebo, but the difference was not statistically significant (52.4% vs. 19.0%, *p* = 0.052). The adverse events of special interest for which the occurrence rates on ES‐481 treatment were at least 5% higher than that on placebo treatment included dizziness (3 [14.3%] vs. 1 [4.8%]), insomnia (3 [14.3%] vs. 0), dysarthria (2 [9.5%] vs. 0), feeling abnormal (2 [9.5%] vs. 0), and gait disturbance (2 [9.5%] vs. 0). Conversely, the treatment‐emergent adverse events for which the occurrence rates on placebo were at least 5% higher than that on ES‐481 treatment included tinnitus (2 [9.5%] placebo vs. 0 ES‐481), upper abdominal pain (2 [9.5%] placebo vs. 0 ES‐481), nausea (2 [9.5%] placebo vs. 0 ES‐481), and lethargy (2 [9.5%] placebo vs. 0 ES‐481). No significant changes in clinical laboratory or vital sign values were noted. Treatment‐emergent adverse event profiles in the double‐blind treatment phase and open‐label extension phase were similar. No new safety signals were noted during the open‐label extension phase. These data are displayed in Tables [Link], [Link].

#### 3.3.3 Pharmacokinetics

After oral administration of ES‐481, ES‐481 plasma concentrations increased rapidly to reach maximum (Tmax) approximately 2 h post‐dose with the concentration of 362.633 ng/ML for 25 mg QD on Day 1/43 and 543.355 ng/ML for 50 mg and 1190.312 ng/ML for 75 mg on Day 28/70, respectively. Median (min, max) of T1/2 was 3.730 (3.59, 3.87) hours, allowing ES‐481 to reach steady‐state prior to the next scheduled dose increase. Mean (SD) CL/F was at around 6.372 (1.5017) L/h and mean (SD) Vz/F was 34.073 (6.2858) L in the 25 mg ES‐481 treatment (*n* = 21) on Day 1/43. An approximately proportional increase in AUC parameters and Cmax was observed for ES‐481 doses 25 mg, 50 mg, and 75 mg. The mean Cmax values increased from 475.618 ng/mL at the 25 mg dose to 1317.042 ng/mL at the 75 mg dose and mean AUC0‐last values increased from 720.011 ng•h/mL at the 25 mg dose to 3384.350 ng•h/mL at the 75 mg dose.

#### 3.3.4 Secondary outcomes

Mean change from baseline in Hamilton anxiety and depression rating scales were similar between ES‐481 and placebo treatment (results summarized in Tables S7 and S8).

### 3.4 Subanalyses

Post‐hoc subanalyses assessed change in self‐reported (diary) seizure frequency on Period 1 data only to avoid the impact of any carry‐over effects. This also demonstrated an anti‐seizure effect of ES‐481 treatment, with at least 44% reduction over the placebo across all treatment weeks. These data are provided in Tables S9 and S10.

### 3.5 Exploratory seizure diary vs. EEG analysis

There was no significant correlation with regard to seizure frequency as measured by seizure diary vs. 4‐h EEG data. Data are displayed in Table S11.

## 4 DISCUSSION

This Phase 2A first‐in‐disease trial found ES‐481 to be safe, well tolerated, and had potential anti‐seizure efficacy at a dose of 75 mg bid compared with placebo, when used as an add‐on ASM for adults with a mixed group of drug‐resistant epilepsies. ES‐481 has a novel, selective mode of action which may help minimize adverse effects while retaining anti‐seizure efficacy, but this will require larger, comparative studies to establish.

Currently available ASMs exert their effects broadly across the CNS, which may contribute to substantial treatment‐related adverse effects. A longitudinal study of 1795 people with newly diagnosed epilepsy reported that nearly 1 in 6 ASMs prescribed were subsequently withdrawn due to intolerable adverse effects, which occurred even at low doses of medication.^15^ This study also found that the rate of intolerable adverse effects with ASM treatment had not changed over the last 30 years, despite a surge in new ASM development.^15^ This reinforces the need for the ongoing development of novel ASMs that focus on tolerability and safety, in addition to seizure control.^15^ ES‐481 treatment offers a potential novel strategy for improving ASM tolerability and safety. ES‐481 is a selective y8‐TARP dependent AMPA inhibitor, and its specific AMPA target is highly expressed in hippocampi and limbic structures involved in epileptogenic networks across a broad range of epilepsies.^16^ Similar targeted approaches are emerging throughout many fields of medicine. So‐called “designer molecules” selectively target pathological substrate while minimizing effects on unaffected tissue to optimize the trade‐off between medication‐related beneficial vs. adverse effects.

ES‐481's mode of action targeting AMPA receptors aligns with that of perampanel, which is a well‐established, effective ASM in widespread clinical use. However, perampanel is a non‐competitive antagonist with a broad effect on AMPA receptors, in contrast to the selective effect of ES‐481 for those containing y8‐TARP. Perampanel has a broad spectrum of anti‐seizure efficacy, approved for clinical use for both focal and generalized epilepsies, and for use as monotherapy or add‐on therapy (approval varies between countries).^17^ A 2022 global pooled analysis of 44 real‐world studies reported that at 12 months, 58.3% of patients on perampanel experienced 50% or greater reduction in seizure frequency from baseline, and a further 23.2% experienced seizure freedom.^17^ Unfortunately, this study also found that 49.9% of people prescribed perampanel reported adverse events at 12 months which included dizziness, somnolence, irritability, and behavioral disorders, and one‐fifth (17.6%) of people needed to discontinue perampanel by 12 months due to adverse events.^17^ Because ES‐481 selectively targets AMPA receptors expressed in the hippocampi and limbic system, in contrast to perampanel that non‐selectively acts on AMPA receptors throughout the CNS, it may potentially provide at least similar anti‐seizure efficacy with an improved safety and tolerability profile. However, this potential advantage will need to be established by larger, comparative trials.

This study uses reduction in seizure frequency relative to baseline as its primary endpoint, measured via the traditional method of patient or carer self‐reported seizure diaries.^18^ Change in seizure frequency measured via patient‐ or carer‐completed seizure diaries is the primary endpoint for most epilepsy clinical trials.^19^ Unfortunately, seizure diary data are frequently inaccurate. Studies have revealed that compared with objective EEG recordings, patients document on average fewer than 50% of their seizures.^19^, ^20^ This may be for several reasons. Patients may not have recognized that a seizure has occurred, they may have impaired ability to document the seizure, or they may forget to record the seizure.^19^ Concerningly, when seizure diary data are inaccurate, it may lead to erroneous conclusions, including false positive and negative trials. With rapid advances in medical engineering and data analysis technology, it is becoming increasingly feasible to perform and accurately quantitate epileptiform activity on high‐quality, prolonged EEG studies. These offer an attractive “objective” measure of EEG activity to complement the “subjective” measures for participants with frequent EEG epileptiform discharges.^21^, ^22^ However, in this trial, our exploratory analysis revealed no significant change in the frequency of occurrence of EEG epileptiform/seizure discharges of >3 s between the therapeutic and placebo arms, and also that these EEG‐detected events did not correlate with the seizure diaries. This may reflect the use of 4‐h normalized EEG epochs in our study, as opposed to longer periods of continuous EEG monitoring or chronic implanted intracranial electrodes used in previous studies.^21^, ^22^ A 2023 scoping review of the role of interictal EEG biomarkers in assessing ASM efficacy suggested this was a promising area, but further research was required to establish its place in clinical epilepsy trials and practice.^23^ Barriers to routine incorporation of EEG efficacy assessments into clinical trials include interrater variability, and further data are needed to understand the timing of EEGs with relation to ASM dosing.^23^ Our study has shown that EEG screening at regular intervals throughout a clinical trial is feasible, but the interpretation of these results, particularly in relation to the seizure diary results and ASM efficacy, will require further study.

### 4.1 Limitations

In keeping with being a first‐in‐disease Phase 2A trial, its primary purpose was to establish dosing and safety profiles, and obtain indicative date of potential efficacy and tolerability with respect to dose. Our sample size was not powered to make conclusive findings on efficacy, and the short time frame (1 week) for quantifying seizures at each dose of ES‐481 (and 4 weeks for the entire treatment period) would increase the chance of under‐ or over‐estimating any treatment effect as there was less time for the natural fluctuations in seizure frequency to even out.^24^ This likely contributes to the high placebo responder rate seen in this trial. In addition, there may have been carry‐over effect from the cross‐over study design. Future Phase 2b/3 trials with longer treatment durations, larger sample sizes, and parallel treatment group design will be needed to more definitely assess the anti‐seizure efficacy of ES‐481. The current study was also not designed to examine treatment response based on epilepsy subtype, but future Phase 3 studies may test whether participants with limbic epilepsy have better responses compared to participants with epilepsy arising from non‐limbic regions. Our study's findings have limited generalizability due to the small cohort size, recruited from a small number of centers. Further studies will also be needed to establish the utility and optimal approach for utilizing epileptiform/seizure discharges on EEG as a surrogate objective biomarker in clinical trials for identifying anti‐seizure efficacy.

## 5 CONCLUSION

This Phase 2A study of ES‐481, a novel potent and selective antagonist of the TARP‐y8‐ dependent receptor, demonstrates that doses of up to 75 mg bid was safe and well tolerated, with preliminary evidence for anti‐seizure effects. This is a promising foundation to inform future, larger, later stage trials of this novel ASM.

## AUTHOR CONTRIBUTIONS

EF—Board‐certified epileptologist, blind reader of study EEGs at the central site, drafted and submitted manuscript. LM—Senior neuroscientist, blind reader of study EEGs at the central site, provided critical revision of manuscript. JG—Senior study coordinator, facilitated participant recruitment and oversaw study workflow, provided critical revision of manuscript. SC—Clinical trials nurse, assessed participants, provided critical revision of manuscript. CR—Neuroscientist, facilitated EEGs, provided critical revision of manuscript. JPN—Board‐certified epileptologist, principal investigator for The Royal Melbourne Hospital study site, screened study EEGs for safety signals, provided critical revision of manuscript. SH—Clinical trials nurse, assessed participants, provided critical revision of manuscript. PP—Board‐certified epileptologist, investigator for Austin Hospital study site, provided critical revision of manuscript. SM—Board‐certified epileptologist, principal investigator for Austin Hospital study site, screened study EEGs for safety signals, provided critical revision of manuscript. HR—Clinical trial coordinator, facilitated patient recruitment, assessed participants, provided critical revision of manuscript. RW—Clinical trial coordinator, facilitated patient recruitment, assessed participants, provided critical revision of manuscript. BW—Clinical trials nurse, assessed participants, provided critical revision of manuscript. DR—Board‐certified epileptologist, principal investigator for Royal Brisbane and Women's Hospital study site, provided critical revision of manuscript. KI—Clinical trials research assistant, facilitated participant recruitment and oversaw study workflow, provided critical revision of manuscript. HZ—Statistician, conducted study statistical analysis, and provided critical revision of manuscript. RN—Chief Scientific Officer of ES Therapeutics, involved in study design, conduct of the study, data interpretation, and critical revision of manuscript. TOB—Board‐certified epileptologist, study chief investigator, and principal investigator of the Alfred Hospital study site, provided critical revision of manuscript.

## FUNDING INFORMATION

This study was sponsored by ES‐Therapeutics LLC. TJOB was funded by his NHMRC Investigator Grants (APP1176426 and APP2034258).

## CONFLICT OF INTEREST STATEMENT

EF/her institution reports grants from Avant Research Foundation, Brain Foundation Australia, Clive and Vera Ramaciotti Foundation (Ramaciotti Health Investment Grant), GPCE, LivaNova (Australia and USA); Lundbeck (Australia); Monash Partners STAR Clinician Fellowship, Monash University Early Career Postdoctoral Fellowship, National Health and Medical Research Council Investigator Grant, Royal Australasian College of Physicians Fellows Research Establishment Fellowship, Sylvia and Charles Viertel Charitable Foundation Clinical Investigator Award, and UCB, outside the submitted work. LM reports no disclosures. JG reports no disclosures. SC reports no disclosures. CR reports no disclosures. JPN reports no disclosures. SH reports no disclosures. PP has received speaker honoraria or consultancy fees to his institution from Chiesi, Eisai, Jazz Pharmaceuticals, LivaNova, Novartis, Sun Pharma, Supernus, and UCB Pharma, outside of the submitted work. He is on the board of the International Registry of Antiepileptic Drugs and Pregnancy (EURAP), a non‐profit organization that has received financial support from Accord, Angelini, Bial, EcuPharma, Eisai, Glenmark, GW Pharma, GlaxoSmithKline, Sanofi, SF Group, Teva, UCB, and Zentiva. He is Deputy Editor for Epilepsia Open. SM reports no disclosures. HR reports no disclosures. RW reports no disclosures. BW reports no disclosures. DR reports no disclosures. KI reports no disclosures. HZ is a paid consultant of ES Therapeutics. RN is a paid consultant of ES Therapeutics and Cyprium Therapeutics. ToB reports research funding to his institution, grants from the NHMRC, MRFF, NIH, and DoD, as well as research funding and consultancies from Industry including UCB Pharma, Eisai Pharma, Kinoxis Pharmaceuticals, Jazz Pharma, LivaNova, and Supernus, outside the submitted work. We confirm that we have read the Journal's position on issues involved in ethical publication and affirm that this report is consistent with those guidelines.

## Supporting information


**Figure S1.** The % improvement in seizure frequency compared with baseline for placebo arms, for Treatment Period 1 vs. Treatment Period 2.

**Figure S2.** The percentage weekly mean seizure frequency improvement compared with baseline during the open‐label extension phase of the trial (up to 38 weeks).

**Table S1.** Change from baseline in weekly seizure frequency in DBT phase.

**Table S2.** Analysis of change from baseline in normalized 4‐h EEG electrographic seizure frequency (i.e., discharges >3 s) during the double‐blind treatment phase.

**Table S3.** Treatment‐emergent adverse events in the double‐blind treatment phase (modified intention‐to‐treat population).

**Table S4.** Summary of treatment‐emergent adverse events by system organ class (double‐blind treatment phase) that occurred in =>5% of the safety population. TEAE = treatment‐emergent adverse event, defined as any adverse event that occurs within the TEAE window. This window starts on the first dosing date and ends 14 days after the last dosing date for non‐serious adverse events and 30 days after the last dosing for serious adverse events. All adverse events that are considered by the investigator as treatment‐related will be treated as TEAEs.

**Table S5.** Summary of treatment‐emergent adverse events by system organ class (double‐blind treatment phase); safety population. TEAE = treatment‐emergent adverse event, defined as any adverse event that occurs within the TEAE window. This window starts on the first dosing date and ends 14 days after the last dosing date for non‐serious adverse events and 30 days after the last dosing for serious adverse events. All adverse events that are considered by the investigator as treatment‐related will be treated as TEAEs.

**Table S6.** Treatment‐emergent adverse events by system organ class and preferred term DBT phase by dosing.

**Table S7.** Change in Hamilton anxiety rating scales for the double‐blind treatment phase compared with baseline (modified intention‐to‐treat population).

**Table S8.** Change in Hamilton depression rating scales for the double‐blind treatment phase (modified intention‐to‐treat population).

**Table S9.** Period 1 change in seizure frequency from baseline in DBT.

**Table S10.** Analysis of weekly seizure frequency in Period 1 of the double‐blind treatment phase.

**Table S11.** Pearson partial correlation coefficients of seizure frequency measured by seizure diaries and by normalized 4‐h EEG data (i.e., epileptiform discharges >3 s).

## Data Availability

The data that support the findings of this study are available on request from the corresponding author. The data are not publicly available due to privacy or ethical restrictions.
