# Supplementary figures and images for: A first in disease trial of the safety, tolerability, and anti‐seizure effects of ES‐481 in drug‐resistant epilepsy
Source: Epilepsia Open. 2026 Jun 18;11(4):1329–42. doi: 10.1002/epi4.70294 (PMC13394730; doi:10.1002/epi4.70294)

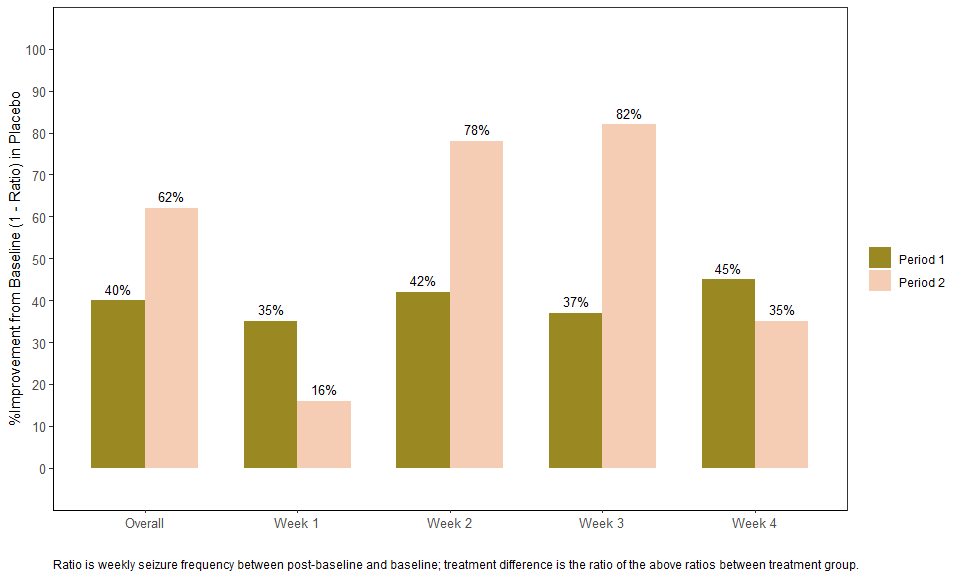

Supplement: Supplementary file 1 — Figure S1. The % improvement in seizure frequency compared with baseline for placebo arms, for Treatment Period 1 vs. Treatment Period 2. [file EPI4-11-1329-s004.tif]

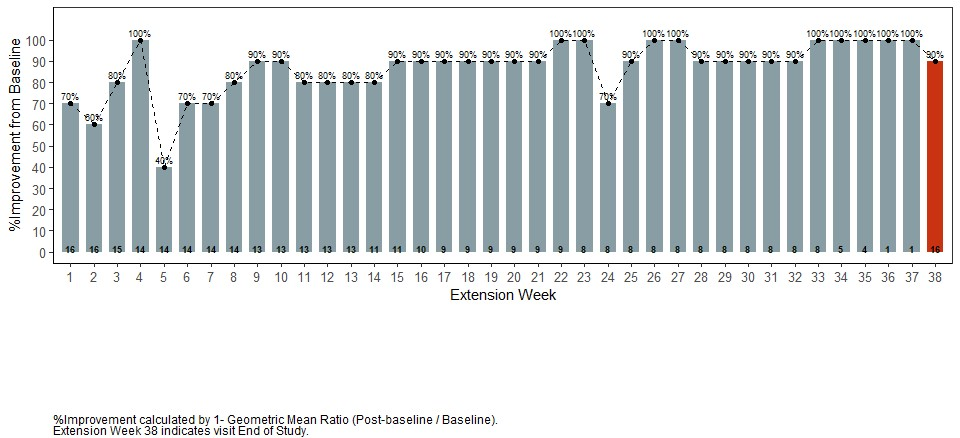

Supplement: Supplementary file 2 — Figure S2. The percentage weekly mean seizure frequency improvement compared with baseline during the open‐label extension phase of the trial (up to 38 weeks). [file EPI4-11-1329-s013.tif]
