# Supplementary material for: A first in disease trial of the safety, tolerability, and anti‐seizure effects of ES‐481 in drug‐resistant epilepsy
Source: Epilepsia Open. 2026 Jun 18;11(4):1329–42. doi: 10.1002/epi4.70294 (PMC13394730; doi:10.1002/epi4.70294)
Supplement: Supplementary file 3 — Table S1. Change from baseline in weekly seizure frequency in DBT phase. [file EPI4-11-1329-s010.docx]

|  |  |  |  | Change from baseline | |
| --- | --- | --- | --- | --- | --- |
| Visit |  | ES-481 N=22 | Placebo N=22 | ES-481 | Placebo |
| Baseline | N | 22 | 22 |  |  |
|  | Mean (SD) | 27.4 (50.1) | 27.4 (50.1) |  |  |
|  | Median | 11.1 | 11.1 |  |  |
|  | Min, Max | 3.5, 242.0 | 3.5, 242.0 |  |  |
|  | | | | | |
| Week 1 | N | 21 | 20 | 21 | 20 |
|  | Mean (SD) | 37.1 (70.6) | 26.0 (50.3) | 8.9 (58.3) | -3.5 (15.2) |
|  | Median | 10.5 | 11.6 | -3.5 | -0.3 |
|  | Min, Max | 0.0, 274.4 | 0.0, 233.3 | -42.0, 241.7 | -44.5, 26.1 |
|  | | | | | |
| Week 2 | N | 21 | 19 | 21 | 19 |
|  | Mean (SD) | 40.6 (95.8) | 27.1 (53.3) | 12.4 (72.1) | -1.3 (14.7) |
|  | Median | 9.3 | 10.5 | -2.6 | 0.7 |
|  | Min, Max | 0.0, 350.0 | 0.0, 237.0 | -39.3, 317.3 | -51.5, 22.3 |
|  | | | | | |
| Week 3 | N | 19 | 19 | 19 | 19 |
|  | Mean (SD) | 21.1 (46.7) | 29.5 (70.3) | -8.1 (12.7) | 1.2 (25.0) |
|  | Median | 7.0 | 9.3 | -3.5 | -1.8 |
|  | Min, Max | 0.0, 210.0 | 0.0, 310.0 | -44.5, 8.2 | -51.5, 68.0 |
|  |  |  |  |  |  |
| Week 4 | N | 19 | 18 | 19 | 18 |
|  | Mean (SD) | 21.1 (38.3) | 17.3 (25.0) | -8.2 (21.6) | -12.3 (31.4) |
|  | Median | 7.0 | 10.2 | -3.5 | -2.2 |
|  | Min, Max | 0.0, 165.7 | 0.0, 114.3 | -76.3, 28.9 | -127.7, 7.7 |

Supplementary Table S1: Change from baseline in weekly seizure frequency in DBT phase
