# Supplementary material for: A first in disease trial of the safety, tolerability, and anti‐seizure effects of ES‐481 in drug‐resistant epilepsy
Source: Epilepsia Open. 2026 Jun 18;11(4):1329–42. doi: 10.1002/epi4.70294 (PMC13394730; doi:10.1002/epi4.70294)
Supplement: Supplementary file 4 — Table S2. Analysis of change from baseline in normalized 4‐h EEG electrographic seizure frequency (i.e., discharges >3 s) during the double‐blind treatment phase. [file EPI4-11-1329-s005.docx]

| Visit |  | ES-481 N=22 | Placebo N=22 | Difference (ES-481 - Placebo) | p-value |
| --- | --- | --- | --- | --- | --- |
| Overall | LSMean (SE) | -2.13 (0.410) | -2.19 (0.302) | 0.06 (0.400) | 0.438 |
|  | 90% CI | (-2.84, -1.42) | (-2.72, -1.67) | (-0.63, 0.76) |  |
|  | | | | | |
| Day 1 | LSMean (SE) | -0.61 (2.995) | -1.67 (0.695) | 2.28 (2.402) | 0.173 |
|  | 90% CI | (-4.39, 5.61) | (-2.84, -0.51) | (-1.73, 6.30) |  |
|  | | | | | |
| Day 8 | LSMean (SE) | -0.77 (0.950) | -2.67 (0.151) | 1.89 (0.970) | 0.028 |
|  | 90% CI | (-2.36, 0.81) | (-2.92, -2.42) | (0.27, 3.51) |  |
|  | | | | | |
| Day 15 | LSMean (SE) | -5.50 (3.349) | -2.00 (0.532) | -3.50 (3.379) | 0.152 |
|  | 90% CI | (-11.09, 0.10) | (-2.89, -1.11) | (-9.14, 2.15) |  |
|  | | | | | |
| Day 22 | LSMean (SE) | -2.39 (0.733) | -1.82 (0.734) | -0.57 (0.582) | 0.165 |
|  | 90% CI | (-3.61, -1.17) | (-3.05, -0.60) | (-1.54, 0.40) |  |
|  | | | | | |
| Day 28 | LSMean (SE) | -2.59 (0.632) | -2.80 (0.123) | 0.21 (0.664) | 0.378 |
|  | 90% CI | (-3.65, -1.54) | (-3.01, -2.60) | (-0.90, 1.32) |  |
|  | | | | | |

Supplementary Table S2: Analysis of change from baseline in normalised 4-hour EEG electrographic seizure frequency (i.e. discharges >3 secs) during the double-blind treatment phase.
