# Supplementary material for: A first in disease trial of the safety, tolerability, and anti‐seizure effects of ES‐481 in drug‐resistant epilepsy
Source: Epilepsia Open. 2026 Jun 18;11(4):1329–42. doi: 10.1002/epi4.70294 (PMC13394730; doi:10.1002/epi4.70294)
Supplement: Supplementary file 5 — Table S3. Treatment‐emergent adverse events in the double‐blind treatment phase (modified intention‐to‐treat population). [file EPI4-11-1329-s002.docx]

|  | ES-481 N=21 | Placebo N=21 | Overall N=22 |
| --- | --- | --- | --- |
| Number of TEAEs | 54 | 52 | 106 |
| Subjects with any TEAEs, n(%) | 18 (85.7) | 16 (76.2) | 20 (90.9) |
| Subjects with serious TEAEs, n(%) | 1 (4.8) | 3 (14.3) | 4 (18.2) |
| Subjects with TEAEs leading to death, n(%) | 0 | 0 | 0 |
| Subjects with TEAEs related to study drug, n(%) | 14 (66.7) | 12 (57.1) | 18 (81.8) |
| Subjects with serious related TEAEs, n(%) | 0 | 2 (9.5) | 2 (9.1) |
| Subjects with TEAEs leading to study drug discontinuation, n(%) | 0 | 4 (19.0) | 4 (18.2) |

Supplementary Table S3: Treatment Emergent Adverse Events in the double-blind treatment phase (modified intention to treat population).
