# Supplementary material for: A first in disease trial of the safety, tolerability, and anti‐seizure effects of ES‐481 in drug‐resistant epilepsy
Source: Epilepsia Open. 2026 Jun 18;11(4):1329–42. doi: 10.1002/epi4.70294 (PMC13394730; doi:10.1002/epi4.70294)
Supplement: Supplementary file 6 — Table S4. Summary of treatment‐emergent adverse events by system organ class (double‐blind treatment phase) that occurred in =>5% of the safety population. TEAE = treatment‐emergent adverse event, defined as any adverse event that occurs within the TEAE window. This window starts on the first dosing date and ends 14 days after the last dosing date for non‐serious adverse events and 30 days after the last dosing for serious adverse events. All adverse events that are considered by the investigator as treatment‐related will be treated as TEAEs. [file EPI4-11-1329-s001.docx]

|  | ES-481 N=21 | | Placebo N=21 | | Overall N=22 | |
| --- | --- | --- | --- | --- | --- | --- |
| System organ class (SOC)  Preferred term (PT) | n (%) | No. of events | n (%) | No. of events | n (%) | No. of events |
| At least one TEAE | 18 (85.7%) | 54 | 16 (76.2) | 52 | 20 (90.9) | 106 |
|  |  |  |  |  |  |  |
| Ear and labyrinth disorders | 0 | 0 | 2 (9.5) | 2 | 2 (9.1) | 2 |
| Tinnitus | 0 | 0 | 2 (9.5) | 2 | 2 (9.1) | 2 |
|  |  |  |  |  |  |  |
| Gastrointestinal disorders | 2 (9.5) | 5 | 5 (23.8) | 6 | 6 (27.3) | 11 |
| Abdominal pain upper | 0 | 0 | 2 (9.5) | 2 | 2 (9.1) | 2 |
| Nausea | 0 | 0 | 2 (9.5) | 2 | 2 (9.1) | 2 |
|  |  |  |  |  |  |  |
| General disorders and administration site conditions | 4 (19.0) | 6 | 3 (14.3) | 3 | 6 (27.3) | 9 |
| Fatigue | 2 (9.5) |  | 2 (9.5) | 2 | 3 (13.6) | 4 |
| Feeling abnormal | 2 (9.5) |  | 0 | 0 | 2 (9.1) | 2 |
| Gait disturbance | 2 (9.5) |  | 0 | 0 | 2 (9.1) | 2 |
|  |  |  |  |  |  |  |
| Injury, poisoning, and postural complications | 4 (19.0) | 4 | 3 (14.3) | 4 | 6 (27.3) | 8 |
| Fall | 4 (19.0) | 4 | 2 (9.5) | 2 | 5 (22.7) | 6 |
|  |  |  |  |  |  |  |
| Nervous system disorders | 9 (42.9) | 18 | 11 (52.4) | 17 | 17 (77.3) | 35 |
| Dizziness | 3 (14.3) | 4 | 1 (4.8) | 1 | 4 (18.2) | 5 |
| Dysarthria | 2 (9.5) | 2 | 0 | 0 | 2 (9.1) | 2 |
| Headache | 1 (4.8) | 1 | 2 (9.5) | 2 | 3 (13.6) | 3 |
| Lethargy | 0 | 0 | 2 (9.5) | 2 | 2 (9.1) | 2 |
| Somnolence | 3 (14.3) | 3 | 2 (9.5) | 3 | 5 (22.7) | 6 |
|  |  |  |  |  |  |  |
| Psychiatric disorders | 5 (23.8) | 9 | 5 (23.8) | 9 | 8 (36.4) | 18 |
| Depressed mood | 1 (4.8) | 1 | 2 (9.5) | 2 | 2 (9.1) | 3 |
| Insomnia | 3 (14.3) | 4 | 0 | 0 | 3 (13.6) | 4 |

Supplementary Table S4: Summary of treatment emergent adverse events by system organ class (double-blind treatment phase), that occurred in =>5% of the safety population. TEAE = Treatment emergent adverse event, defined as any adverse event that occurs within the TEAE window. This window starts on the first dosing date and ends 14 days after the last dosing date for non-serious adverse events and 30 days after the last dosing for serious adverse events. All adverse events that are considered by the Investigator as treatment related will be treated as TEAEs.

Patients are counted only once for each primary SOC and/or each preferred term.

Participant ES-481-C201-002-003 was excluded from the placebo treatment group as the participant discontinued the study before taking any placebo treatment in the double-blinded period. Participant ES-481-C201-003-001 was excluded from the ES-481 treatment group as the participant discontinued the study before taking any ES-481 treatment in the double-blinded period.
