# Supplementary material for: A first in disease trial of the safety, tolerability, and anti‐seizure effects of ES‐481 in drug‐resistant epilepsy
Source: Epilepsia Open. 2026 Jun 18;11(4):1329–42. doi: 10.1002/epi4.70294 (PMC13394730; doi:10.1002/epi4.70294)
Supplement: Supplementary file 7 — Table S5. Summary of treatment‐emergent adverse events by system organ class (double‐blind treatment phase); safety population. TEAE = treatment‐emergent adverse event, defined as any adverse event that occurs within the TEAE window. This window starts on the first dosing date and ends 14 days after the last dosing date for non‐serious adverse events and 30 days after the last dosing for serious adverse events. All adverse events that are considered by the investigator as treatment‐related will be treated as TEAEs. [file EPI4-11-1329-s006.docx]

|  | ES-481 N=21 | | Placebo N=21 | | Overall N=22 | | |
| --- | --- | --- | --- | --- | --- | --- | --- |
| System organ class (SOC)  Preferred term (PT) | n (%) | No. of events | n (%) | No. of events | n (%) | No. of events |  |
| At least one TEAE | 18 (85.7%) | 54 | 16 (76.2) | 52 | 20 (90.9) | 106 |  |
|  |  |  |  |  |  |  |  |
| Ear and labyrinth disorders | 0 | 0 | 2 (9.5) | 2 | 2 (9.1) | 2 |  |
| Tinnitus | 0 | 0 | 2 (9.5) | 2 | 2 (9.1) | 2 |  |
|  |  |  |  |  |  |  |  |
| Eye disorders | 2 (9.5) | 2 | 0 | 0 | 2 (9.1) | 2 |  |
| Diplopia | 1 (4.8) | 1 | 0 | 0 | 1 (4.5) | 1 |  |
| Vision blurred | 1 (4.8) | 1 | 0 | 0 | 1 (4.5) | 1 |  |
|  |  |  |  |  |  |  |  |
| Gastrointestinal disorders | 2 (9.5) | 5 | 5 (23.8) | 6 | 6 (27.3) | 11 |  |
| Abdominal pain | 1 (4.8) | 1 | 0 | 0 | 1 (4.5) | 1 |  |
| Abdominal pain upper | 0 | 0 | 2 (9.5) | 2 | 2 (9.1) | 2 |  |
| Diarrhoea | 1 (4.8) | 2 | 1 (4.8) | 1 | 2 (9.1) | 3 |  |
| Eructation | 1 (4.8) | 1 | 0 | 0 | 1 (4.5) | 1 |  |
| Hypoaesthesia oral | 1 (4.8) | 1 | 0 | 0 | 1 (4.5) | 1 |  |
| Nausea | 0 | 0 | 2 (9.5) | 2 | 2 (9.1) | 2 |  |
| Toothache | 0 | 0 | 1 (4.8) | 1 | 1 (4.5) | 1 |  |

(continues next page)

Supplementary Table S5 (continued)

|  | ES-481 N=21 | | Placebo N=21 | | Overall N=22 | | |
| --- | --- | --- | --- | --- | --- | --- | --- |
| System organ class (SOC)  Preferred term (PT) | n (%) | No. of events | n (%) | No. of events | n (%) | No. of events |  |
| General disorders and administration site conditions | 4 (19.0) | 6 | 3 (14.3) | 3 | 6 (27.3) | 9 |  |
| Fatigue | 2 (9.5) |  | 2 (9.5) | 2 | 3 (13.6) | 4 |  |
| Feeling abnormal | 2 (9.5) |  | 0 | 0 | 2 (9.1) | 2 |  |
| Gait disturbance | 2 (9.5) |  | 0 | 0 | 2 (9.1) | 2 |  |
| Vaccination site reaction | 0 | 0 | 1 (4.8) | 1 | 1 (4.5) | 1 |  |
|  |  |  |  |  |  |  |  |
| Infections and infestations | 2 (9.5) | 2 | 4 (19.0) | 5 | 5 (22.7) | 7 |  |
| Conjunctivitis | 1 (4.8) | 1 | 1 (4.8) | 1 | 2 (9.1) | 2 |  |
| Gastroenteritis | 0 | 0 | 1 (4.8) | 1 | 1 (4.5) | 1 |  |
| Nasopharyngitis | 1 (4.8) | 1 | 1 (4.8) | 1 | 2 (9.1) | 2 |  |
| Oral candidiasis | 0 | 0 | 1 (4.8) | 1 | 1 (4.5) | 1 |  |
| Tinea infection | 0 | 0 | 1 (4.8) | 1 | 1 (4.5) | 1 |  |
|  |  |  |  |  |  |  |  |
| Injury, poisoning, and postural complications | 4 (19.0) | 4 | 3 (14.3) | 4 | 6 (27.3) | 8 |  |
| Fall | 4 (19.0) | 4 | 2 (9.5) | 2 | 5 (22.7) | 6 |  |
| Skin laceration | 0 | 0 | 1 (4.8) | 1 | 1 (4.5) | 1 |  |
| Tongue injury | 0 | 0 | 1 (4.8) | 1 | 1 (4.5) | 1 |  |

(continues next page)

Supplementary Table S5 (continued)

|  | ES-481 N=21 | | Placebo N=21 | | Overall N=22 | | |
| --- | --- | --- | --- | --- | --- | --- | --- |
| System organ class (SOC)  Preferred term (PT) | n (%) | No. of events | n (%) | No. of events | n (%) | No. of events |  |
| Investigations | 1 (4.8) | 1 | 1 (4.8) | 1 | 2 (9.1) | 2 |  |
| Blood creatine phosphokinase increased | 1 (4.8) | 1 | 0 | 0 | 1 (4.5) | 1 |  |
| Liver function test abnormal | 0 | 0 | 1 (4.8) | 1 | 1 (4.5) | 1 |  |
|  |  |  |  |  |  |  |  |
| Metabolism and nutrition disorders | 0 | 0 | 1 (4.8) | 1 | 1 (4.5) | 1 |  |
| Decreased appetite | 0 | 0 | 1 (4.8) | 1 | 1 (4.5) | 1 |  |
|  |  |  |  |  |  |  |  |
| Musculoskeletal and connective tissue disorders | 2 (9.5) | 2 | 1 (4.8) | 1 | 3 (13.6) | 3 |  |
| Back pain | 1 (4.8) | 1 | 0 | 0 | 1 (4.5) | 1 |  |
| Myalgia | 0 | 0 | 1 (4.8) | 1 | 1 (4.5) | 1 |  |
| Neck pain | 1 (4.8) | 1 | 0 | 0 | 1 (4.5) | 1 |  |

(continues next page)

Supplementary Table S5 (continued)

|  | ES-481 N=21 | | Placebo N=21 | | Overall N=22 | | |
| --- | --- | --- | --- | --- | --- | --- | --- |
| System organ class (SOC)  Preferred term (PT) | n (%) | No. of events | n (%) | No. of events | n (%) | No. of events |  |
| Nervous system disorders | 9 (42.9) | 18 | 11 (52.4) | 17 | 17 (77.3) | 35 |  |
| Aphasia | 1 (4.8) | 1 | 0 | 0 | 1 (4.5) | 1 |  |
| Balance disorder | 0 | 0 | 1 (4.8) | 1 | 1 (4.5) | 1 |  |
| Dizziness | 3 (14.3) | 4 | 1 (4.8) | 1 | 4 (18.2) | 5 |  |
| Dysarthria | 2 (9.5) | 2 | 0 | 0 | 2 (9.1) | 2 |  |
| Dyskinesia | 0 | 0 | 1 (4.8) | 1 | 1 (4.5) | 1 |  |
| Fine motor skill dysfunction | 1 (4.8) | 1 | 0 | 0 | 1 (4.5) | 1 |  |
| Headache | 1 (4.8) | 1 | 2 (9.5) | 2 | 3 (13.6) | 3 |  |
| Hypoaesthesia | 1 (4.8) | 1 | 0 | 0 | 1 (4.5) | 1 |  |
| Lethargy | 0 | 0 | 2 (9.5) | 2 | 2 (9.1) | 2 |  |
| Memory impairment | 1 (4.8) | 1 | 1 (4.8) | 1 | 2 (9.1) | 2 |  |
| Migraine | 1 (4.8) | 1 | 0 | 0 | 1 (4.5) | 1 |  |
| Paraesthesia | 1 (4.8) | 1 | 1 (4.8) | 1 | 2 (9.1) | 2 |  |
| Postictal state | 1 (4.8) | 1 | 0 | 0 | 1 (4.5) | 1 |  |
| Sciatica | 0 | 0 | 1 (4.8) | 1 | 1 (4.5) | 1 |  |
| Seizure | 1 (4.8) | 1 | 1 (4.8) | 1 | 2 (9.1) | 2 |  |
| Somnolence | 3 (14.3) | 3 | 2 (9.5) | 3 | 5 (22.7) | 6 |  |
| Speech disorder | 0 | 0 | 1 (4.8) | 1 | 1 (4.5) | 1 |  |
| Syncope | 0 | 0 | 1 (4.8) | 1 | 1 (4.5) | 1 |  |
| Tremor | 0 | 0 | 1 (4.8) | 1 | 1 (4.5) | 1 |  |

(continues next page)

Supplementary Table S5 (continued)

|  | ES-481 N=21 | | Placebo N=21 | | Overall N=22 | | |
| --- | --- | --- | --- | --- | --- | --- | --- |
| System organ class (SOC)  Preferred term (PT) | n (%) | No. of events | n (%) | No. of events | n (%) | No. of events |  |
| Psychiatric disorders | 5 (23.8) | 9 | 5 (23.8) | 9 | 8 (36.4) | 18 |  |
| Agitation | 0 | 0 | 1 (4.8) | 1 | 1 (4.5) | 1 |  |
| Anxiety | 1 (4.8) | 1 | 0 | 0 | 1 (4.5) | 1 |  |
| Bradyphrenia | 0 | 0 | 1 (4.8) | 1 | 1 (4.5) | 1 |  |
| Depressed mood | 1 (4.8) | 1 | 2 (9.5) | 2 | 2 (9.1) | 3 |  |
| Depression | 1 (4.8) | 1 | 0 | 0 | 1 (4.5) | 1 |  |
| Initial insomnia | 0 | 0 | 1 (4.8) | 1 | 1 (4.5) | 1 |  |
| Insomnia | 3 (14.3) | 4 | 0 | 0 | 3 (13.6) | 4 |  |
| Irritability | 1 (4.8) | 1 | 1 (4.8) | 3 | 2 (9.1) | 4 |  |
| Mood swings | 1 (4.8) | 1 | 1 (4.8) | 1 | 1 (4.5) | 2 |  |
|  |  |  |  |  |  |  |  |
| Renal and urinary disorders | 1 (4.8) | 1 | 0 | 0 | 1 (4.5) | 1 |  |
| Pollakiuria | 1 (4.8) | 1 | 0 | 0 | 1 (4.5) | 1 |  |
|  |  |  |  |  |  |  |  |
| Respiratory, thoracic and mediastinal disorders | 1 (4.8) | 1 | 0 | 0 | 1 (4.5) | 1 |  |
| Throat irritation | 1 (4.8) | 1 | 0 | 0 | 1 (4.5) | 1 |  |

(continues next page)

Supplementary Table S5 (continued)

|  | ES-481 N=21 | | Placebo N=21 | | Overall N=22 | | |
| --- | --- | --- | --- | --- | --- | --- | --- |
| System organ class (SOC)  Preferred term (PT) | n (%) | No. of events | n (%) | No. of events | n (%) | No. of events |  |
| Skin and subcutaneous tissue disorders | 3 (14.3) | 3 | 2 (9.5) | 3 | 5 (22.7) | 6 |  |
| Dermatitis contact | 1 (4.8) | 1 | 0 | 0 | 1 (4.5) | 1 |  |
| Hidradenitis | 0 | 0 | 1 (4.8) | 1 | 1 (4.5) | 1 |  |
| Night sweats | 1 (4.8) | 1 | 0 | 0 | 1 (4.5) | 1 |  |
| Rash | 1 (4.8) | 1 | 1 (4.8) | 1 | 2 (9.1) | 2 |  |
| Rash pruritic | 0 | 0 | 1 (4.8) | 1 | 1 (4.5) | 1 |  |

Supplementary Table S5: Summary of treatment emergent adverse events by system organ class (double-blind treatment phase); safety population. TEAE = Treatment emergent adverse event, defined as any adverse event that occurs within the TEAE window. This window starts on the first dosing date and ends 14 days after the last dosing date for non-serious adverse events and 30 days after the last dosing for serious adverse events. All adverse events that are considered by the Investigator as treatment related will be treated as TEAEs.

Patients are counted only once for each primary SOC and/or each preferred term.

Participant ES-481-C201-002-003 was excluded from the placebo treatment group as the participant discontinued the study before taking any placebo treatment in the double-blinded period. Participant ES-481-C201-003-001 was excluded from the ES-481 treatment group as the participant discontinued the study before taking any ES-4481 treatment in the double-blinded period.
