# Supplementary material for: A first in disease trial of the safety, tolerability, and anti‐seizure effects of ES‐481 in drug‐resistant epilepsy
Source: Epilepsia Open. 2026 Jun 18;11(4):1329–42. doi: 10.1002/epi4.70294 (PMC13394730; doi:10.1002/epi4.70294)
Supplement: Supplementary file 8 — Table S6. Treatment‐emergent adverse events by system organ class and preferred term DBT phase by dosing. [file EPI4-11-1329-s008.docx]

| ES-481 | | | | | | | |
| --- | --- | --- | --- | --- | --- | --- | --- |
| SYSTEM ORGAN CLASS (SOC)  PREFERRED TERM (PT) | 25 mg N=21 n (%) E |  | 50 mg N=20 n (%) E |  | 100 mg N=19 n (%) E |  | 150 mg N=18 n (%) E |
| At least one TEAE | 6 (28.6) 7 |  | 9 (45.0) 10 |  | 11 (57.9) 18 |  | 8 (44.4) 17 |
|  | | | | | | | |
| Eye disorders | 0 |  | 1 (5.0) 1 |  | 0 |  | 1 (5.6) 1 |
|  | | | | | | | |
| Gastrointestinal disorders | 1 (4.8) 1 |  | 0 |  | 0 |  | 2 (11.1) 4 |
|  | | | | | | | |
| General disorders and administration site conditions | 1 (4.8) 1 |  | 1 (5.0) 1 |  | 0 |  | 3 (16.7) 4 |
|  | | | | | | | |
| Infections and infestations | 1 (4.8) 1 |  | 1 (5.0) 1 |  | 0 |  | 0 |
|  | | | | | | | |
| Injury, poisoning and procedural complications | 0 |  | 1 (5.0) 1 |  | 2 (10.5) 2 |  | 1 (5.6) 1 |
|  | | | | | | | |
| Investigations | 0 |  | 0 |  | 1 (5.3) 1 |  | 0 |
|  | | | | | | | |
| Diplopia | 0 |  | 0 |  | 0 |  | 1 (5.6) 1 |
| Vision blurred | 0 |  | 1 (5.0) 1 |  | 0 |  | 0 |
|  | | | | | | | |
| Abdominal pain | 0 |  | 0 |  | 0 |  | 1 (5.6) 1 |
| Diarrhoea | 1 (4.8) 1 |  | 0 |  | 0 |  | 1 (5.6) 1 |
| Eructation | 0 |  | 0 |  | 0 |  | 1 (5.6) 1 |
| Hypoaesthesia oral | 0 |  | 0 |  | 0 |  | 1 (5.6) 1 |
|  | | | | | | | |
| Fatigue | 1 (4.8) 1 |  | 0 |  | 0 |  | 1 (5.6) 1 |
| Feeling abnormal | 0 |  | 0 |  | 0 |  | 2 (11.1) 2 |
| Gait disturbance | 0 |  | 1 (5.0) 1 |  | 0 |  | 1 (5.6) 1 |
|  | | | | | | | |
| Conjunctivitis | 1 (4.8) 1 |  | 0 |  | 0 |  | 0 |
| Nasopharyngitis | 0 |  | 1 (5.0) 1 |  | 0 |  | 0 |
|  | | | | | | | |
| Fall | 0 |  | 1 (5.0) 1 |  | 2 (10.5) 2 |  | 1 (5.6) 1 |
|  | | | | | | | |
| Blood creatine phosphokinase increased | 0 |  | 0 |  | 1 (5.3) 1 |  | 0 |
|  | | | | | | | |

| ES-481 | | | | | | | |
| --- | --- | --- | --- | --- | --- | --- | --- |
| SYSTEM ORGAN CLASS (SOC)  PREFERRED TERM (PT) | 25 mg N=21 n (%) E |  | 50 mg N=20 n (%) E |  | 100 mg N=19 n (%) E |  | 150 mg N=18 n (%) E |
| Musculoskeletal and connective tissue disorders | 1 (4.8) 1 |  | 0 |  | 0 |  | 1 (5.6) 1 |
|  | | | | | | | |
| Nervous system disorders | 2 (9.5) 2 |  | 1 (5.0) 1 |  | 9 (47.4) 11 |  | 2 (11.1) 2 |
|  | | | | | | | |
| Psychiatric disorders | 0 |  | 4 (20.0) 5 |  | 1 (5.3) 2 |  | 1 (5.6) 1 |
|  | | | | | | | |
| Back pain | 0 |  | 0 |  | 0 |  | 1 (5.6) 2 |
| Neck pain | 1 (4.8) 1 |  | 0 |  | 0 |  | 0 |
|  | | | | | | | |
| Aphasia | 1 (4.8) 1 |  | 0 |  | 0 |  | 0 |
| Dizziness | 0 |  | 0 |  | 2 (10.5) 2 |  | 1 (5.6) 1 |
| Dysarthria | 0 |  | 0 |  | 2 (10.5) 2 |  | 0 |
| Fine motor skill dysfunction | 0 |  | 0 |  | 1 (5.3) 1 |  | 0 |
| Headache | 0 |  | 0 |  | 1 (5.3) 1 |  | 0 |
| Hypoaesthesia | 0 |  | 0 |  | 0 |  | 1 (5.6) 1 |
| Memory impairment | 1 (4.8) 1 |  | 0 |  | 0 |  | 0 |
| Migraine | 0 |  | 1 (5.0) 1 |  | 0 |  | 0 |
| Paraesthesia | 0 |  | 0 |  | 1 (5.3) 1 |  | 0 |
| Postictal state | 0 |  | 0 |  | 0 |  | 0 |
| Seizure | 0 |  | 0 |  | 1 (5.3) 1 |  | 0 |
| Somnolence | 0 |  | 0 |  | 3 (15.8) 3 |  | 0 |
|  | | | | | | | |
| Anxiety | 0 |  | 0 |  | 1 (5.3) 1 |  | 0 |
| Depressed mood | 0 |  | 1 (5.0) 1 |  | 0 |  | 0 |
| Depression | 0 |  | 0 |  | 0 |  | 1 (5.6) 1 |

|  | | | | ES-481 | | | |
| --- | --- | --- | --- | --- | --- | --- | --- |
| SYSTEM ORGAN CLASS (SOC)  PREFERRED TERM (PT) | 25 mg N=21 n (%) E |  | 50 mg N=20 n (%) E |  | 100 mg N=19 n (%) E |  | 150 mg N=18 n (%) E |
| Renal and urinary disorders | 1 (4.8) 1 |  | 0 |  | 0 |  | 0 |
|  | | | | | | | |
| Respiratory, thoracic and mediastinal disorders | 0 |  | 0 |  | 0 |  | 1 (5.6) 1 |
|  | | | | | | | |
| Skin and subcutaneous tissue disorders | 0 |  | 0 |  | 2 (10.5) 2 |  | 1 (5.6) 1 |
|  | | | | | | | |
| Insomnia | 0 |  | 2 (10.0) 2 |  | 1 (5.3) 1 |  | 1 (5.6) 1 |
| Irritability | 0 |  | 1 (5.0) 1 |  | 0 |  | 0 |
| Mood swings | 0 |  | 1 (5.0) 1 |  | 0 |  | 0 |
|  | | | | | | | |
| Pollakiuria | 1 (4.8) 1 |  | 0 |  | 0 |  | 0 |
|  | | | | | | | |
| Throat irritation | 0 |  | 0 |  | 0 |  | 1 (5.6) 1 |
|  | | | | | | | |
| Dermatitis contact | 0 |  | 0 |  | 0 |  | 1 (5.6) 1 |
| Night sweats | 0 |  | 0 |  | 1 (5.3) 1 |  | 0 |
| Rash | 0 |  | 0 |  | 1 (5.3) 1 |  | 0 |
|  | | | | | | | |

Supplementary table S6: Treatment emergent adverse events by system organ class and preferred term DBT phase by dosing.

N (%) E: Number and percentage of subjects within the dosing group, and the number of events within the dosing group. A treatment emergent adverse event is defined as any adverse event that occurs within the treatment emergent adverse event window. This window starts on the first dosing date and ends 14 days after the last dosing date for non-serious adverse events, and 30 days after the last dosing for serious adverse events. All adverse events that are considered by the Investigator as treatment related are treated as treatment emergent adverse events. Patients are only counted once for each primary system organ class and/or each preferred term. All treatment emergent adverse events are summarized by dosing of adverse events onset and a subject may be in more than one group.
