# Supplementary material for: A first in disease trial of the safety, tolerability, and anti‐seizure effects of ES‐481 in drug‐resistant epilepsy
Source: Epilepsia Open. 2026 Jun 18;11(4):1329–42. doi: 10.1002/epi4.70294 (PMC13394730; doi:10.1002/epi4.70294)
Supplement: Supplementary file 9 — Table S7. Change in Hamilton anxiety rating scales for the double‐blind treatment phase compared with baseline (modified intention‐to‐treat population). [file EPI4-11-1329-s011.docx]

| Visit |  | ES-481 N=22 | Placebo N=22 | Difference (ES-481 - Placebo) | p-value |
| --- | --- | --- | --- | --- | --- |
| Overall | LSMean (SE) | -3.92 (0.936) | -3.88 (1.134) | -0.05 (1.164) | 0.485 |
|  | 90% CI | (-5.54, -2.31) | (-5.84, -1.92) | (-2.06, 1.97) |  |
|  | | | | | |
| Day 1 | LSMean (SE) | -3.52 (1.389) | -2.53 (1.134) | -0.99 (1.532) | 0.261 |
|  | 90% CI | (-5.85, -1.19) | (-4.44, -0.63) | (-3.56, 1.58) |  |
|  | | | | | |
| Day 8 | LSMean (SE) | -5.08 (1.056) | -3.46 (1.152) | -1.62 (1.580) | 0.155 |
|  | 90% CI | (-6.86, -3.31) | (-5.40, -1.53) | (-4.27, 1.03) |  |
|  | | | | | |
| Day 15 | LSMean (SE) | -4.36 (1.412) | -5.15 (1.200) | 0.79 (1.775) | 0.330 |
|  | 90% CI | (-6.73, -1.99) | (-7.16, -3.13) | (-2.19, 3.76) |  |
|  | | | | | |
| Day 22 | LSMean (SE) | -4.77 (1.305) | -4.79 (1.733) | 0.03 (2.059) | 0.495 |
|  | 90% CI | (-6.95, -2.58) | (-7.70, -1.89) | (-3.43, 3.48) |  |
|  | | | | | |
| Day 28 | LSMean (SE) | -1.88 (2.845) | -3.45 (2.610) | 1.57 (3.436) | 0.325 |
|  | 90% CI | (-6.66, 2.89) | (-7.83, 0.93) | (-4.20, 7.33) |  |
|  | | | | | |

Supplementary Table S7: Change in Hamilton anxiety rating scales for the double-blind treatment phase compared with baseline (modified intention to treat population).
