# Supplementary material for: A first in disease trial of the safety, tolerability, and anti‐seizure effects of ES‐481 in drug‐resistant epilepsy
Source: Epilepsia Open. 2026 Jun 18;11(4):1329–42. doi: 10.1002/epi4.70294 (PMC13394730; doi:10.1002/epi4.70294)
Supplement: Supplementary file 10 — Table S8. Change in Hamilton depression rating scales for the double‐blind treatment phase (modified intention‐to‐treat population). [file EPI4-11-1329-s012.docx]

| Visit |  | ES-481 N=22 | Placebo N=22 | Difference (ES-481 - Placebo) | p-value |
| --- | --- | --- | --- | --- | --- |
| Overall | LSMean (SE) | -1.25 (0.593) | -1.10 (0.845) | -0.15 (0.737) | 0.421 |
|  | 90% CI | (-2.28, -0.23) | (-2.56, 0.36) | (-1.42, 1.12) |  |
|  | | | | | |
| Day 1 | LSMean (SE) | -0.99 (1.077) | -0.10 (0.805) | -0.89 (1.313) | 0.250 |
|  | 90% CI | (-2.80, 0.82) | (-1.45, 1.25) | (-3.10, 1.32) |  |
|  | | | | | |
| Day 8 | LSMean (SE) | -2.63 (0.653) | -0.80 (0.797) | -1.83 (1.156) | 0.060 |
|  | 90% CI | (-3.73, -1.54) | (-2.14, 0.54) | (-3.77, 0.11) |  |
|  | | | | | |
| Day 15 | LSMean (SE) | -1.90 (0.818) | -1.07 (1.093) | -0.83 (0.970) | 0.198 |
|  | 90% CI | (-3.28, -0.53) | (-2.91, 0.77) | (-2.46, 0.80) |  |
|  | | | | | |
| Day 22 | LSMean (SE) | -2.33 (0.583) | -1.40 (1.223) | -0.93 (1.326) | 0.245 |
|  | 90% CI | (-3.31, -1.35) | (-3.46, 0.65) | (-3.15, 1.30) |  |
|  | | | | | |
| Day 28 | LSMean (SE) | 1.59 (1.996) | -2.14 (2.236) | 3.73 (2.501) | 0.072 |
|  | 90% CI | (-1.77, 4.94) | (-5.90, 1.62) | (-0.47, 7.94) |  |
|  | | | | | |

Supplementary Table S8: Change in Hamilton depression rating scales for the double-blind treatment phase (modified intention to treat population).
