# Supplementary material for: A first in disease trial of the safety, tolerability, and anti‐seizure effects of ES‐481 in drug‐resistant epilepsy
Source: Epilepsia Open. 2026 Jun 18;11(4):1329–42. doi: 10.1002/epi4.70294 (PMC13394730; doi:10.1002/epi4.70294)
Supplement: Supplementary file 11 — Table S9. Period 1 change in seizure frequency from baseline in DBT. [file EPI4-11-1329-s003.docx]

| Visit |  | ES-481 N=11 | Placebo N=11 | Difference (ES-481 - Placebo) | p-value |
| --- | --- | --- | --- | --- | --- |
| Overall | LSMean of Log-transformed Score (SE) | -1.24 (0.661) | -0.51 (0.452) | -0.73 (0.867) | 0.204 |
|  | Ratio^a^ | 0.29 | 0.60 | 0.48 |  |
|  | 90% CI | (0.09, 0.91) | (0.28, 1.31) | (0.11, 2.15) |  |
|  | | | | | |
| Week 1 | LSMean of Log-transformed Score (SE) | -1.20 (0.733) | -0.43 (0.577) | -0.77 (0.993) | 0.220 |
|  | Ratio^a^ | 0.30 | 0.65 | 0.46 |  |
|  | 90% CI | (0.09, 1.03) | (0.25, 1.71) | (0.09, 2.43) |  |
|  | | | | | |
| Week 2 | LSMean of Log-transformed Score (SE) | -1.36 (0.673) | -0.54 (0.657) | -0.82 (1.005) | 0.210 |
|  | Ratio^a^ | 0.26 | 0.58 | 0.44 |  |
|  | 90% CI | (0.08, 0.79) | (0.19, 1.75) | (0.08, 2.37) |  |
|  | | | | | |
| Week 3 | LSMean of Log-transformed Score (SE) | -1.22 (0.672) | -0.46 (0.735) | -0.76 (1.052) | 0.238 |
|  | Ratio^a^ | 0.29 | 0.63 | 0.47 |  |
|  | 90% CI | (0.10, 0.91) | (0.18, 2.15) | (0.08, 2.73) |  |
|  | | | | | |
| Week 4 | LSMean of Log-transformed Score (SE) | -1.18 (0.678) | -0.60 (0.623) | -0.58 (0.963) | 0.273 |
|  | Ratio^a^ | 0.31 | 0.55 | 0.56 |  |
|  | 90% CI | (0.10, 0.95) | (0.19, 1.56) | (0.11, 2.79) |  |
|  | | | | | |

Supplementary Table S9: Period 1 change in seizure frequency from baseline in DBT
