# Supplementary material for: A first in disease trial of the safety, tolerability, and anti‐seizure effects of ES‐481 in drug‐resistant epilepsy
Source: Epilepsia Open. 2026 Jun 18;11(4):1329–42. doi: 10.1002/epi4.70294 (PMC13394730; doi:10.1002/epi4.70294)
Supplement: Supplementary file 12 — Table S10. Analysis of weekly seizure frequency in Period 1 of the double‐blind treatment phase. [file EPI4-11-1329-s007.docx]

| **Seizure Frequency Responder** |  | **ES-481**  **N=11** | **Placebo**  **N=11** | **p-value** |
| --- | --- | --- | --- | --- |
| >=30% Decrease from baseline | Responder | 8 (72.7) | 4 (36.4) | 0.099 |
|  | Non-responder | 3 (27.3) | 7 (63.6) |  |
| >=50% Decrease from Baseline | Responder | 4 (36.4) | 2 (18.2) | 0.318 |
|  | Non-responder | 7 (63.6) | 9 (81.8) |  |
| One week complete remission | Responder | 2 (18.2) | 2 (18.2) | 0.500 |
|  | Non-responder | 9 (81.8) | 9 (81.8) |  |

Supplementary Table S10: Analysis of weekly seizure frequency in Period 1 of the double-blind treatment phase.

Complete remission means 0 weekly seizure frequency during any of the treatment weeks.
