# Supplementary material for: A first in disease trial of the safety, tolerability, and anti‐seizure effects of ES‐481 in drug‐resistant epilepsy
Source: Epilepsia Open. 2026 Jun 18;11(4):1329–42. doi: 10.1002/epi4.70294 (PMC13394730; doi:10.1002/epi4.70294)
Supplement: Supplementary file 13 — Table S11. Pearson partial correlation coefficients of seizure frequency measured by seizure diaries and by normalized 4‐h EEG data (i.e., epileptiform discharges >3 s). [file EPI4-11-1329-s009.docx]

| Visit |  | ES-481 | Placebo | Overall |
| --- | --- | --- | --- | --- |
| Day 1 | Pearson’s correlation coefficient | -0.160 | 0.508 | -0.035 |
|  | p-value | 0.525 | 0.026 | 0.831 |
|  | N | 19 | 20 | 20 |
|  |  |  |  |  |
| Day 8 | Pearson’s correlation coefficient | -0.079 | -0.260 | -0.035 |
|  | p-value | 0.747 | 0.297 | 0.835 |
|  | N | 20 | 19 | 21 |
|  |  |  |  |  |
| Day 15 | Pearson’s correlation coefficient | 0.500 | -0.165 | 0.013 |
|  | p-value | 0.041 | 0.528 | 0.939 |
|  | N | 18 | 18 | 20 |
|  |  |  |  |  |
| Day 22 | Pearson’s correlation coefficient | 0.097 | -0.217 | 0.062 |
|  | p-value | 0.711 | 0.403 | 0.718 |
|  | N | 18 | 18 | 20 |
|  |  |  |  |  |
| Day 28 | Pearson’s correlation coefficient | 0.233 | -0.128 | 0.283 |
|  | p-value | 0.351 | 0.650 | 0.100 |
|  | N | 19 | 16 | 19 |

Supplementary Table S11: Pearson partial correlation coefficients of seizure frequency measured by seizure diaries and by normalised 4-hour EEG data (i.e. epileptiform discharges >3 seconds).
